# Immune Profile of Honduran Schoolchildren with Intestinal Parasites: The Skewed Response against Geohelminths

**DOI:** 10.1155/2016/1769585

**Published:** 2016-11-01

**Authors:** José Antonio Gabrie, María Mercedes Rueda, Carol Anahelka Rodríguez, Maritza Canales, Ana Lourdes Sanchez

**Affiliations:** ^1^Department of Health Sciences, Brock University, St. Catharines, ON, Canada; ^2^School of Microbiology, National Autonomous University of Honduras (UNAH), Tegucigalpa, Honduras; ^3^Microbiology Research Institute, National Autonomous University of Honduras (UNAH), Tegucigalpa, Honduras

## Abstract

Soil-transmitted helminth infections typically induce a type-2 immune response (Th2), but no immunoepidemiological studies have been undertaken in Honduras, an endemic country where the main control strategy is children's annual deworming. We aimed to characterize the immune profile of Honduran schoolchildren harbouring these parasitoses. Demographic and epidemiological data were obtained through a survey; nutritional status was assessed through anthropometry; intestinal parasites were diagnosed by formol-ether and Kato-Katz; and blood samples were collected to determine immunological markers including Th1/Th2 cytokines, IgE, and eosinophil levels. A total of 225 children participated in the study, all of whom had received deworming during the national campaign five months prior to the study. Trichuriasis and ascariasis prevalence were 22.2% and 20.4%, respectively. Stunting was associated with both age and trichuriasis, whereas ascariasis was associated with sex and household conditions. Helminth infections were strongly associated with eosinophilia and hyper-IgE as well as with a Th2-polarized response (increased levels of IL-13, IL-10, and IL4/IFN-*γ* ratios and decreased levels of IFN-*γ*). Pathogenic protozoa infections were associated with a Th1 response characterized by elevated levels of IFN-*γ* and decreased IL10/IFN-*γ* ratios. Even at low prevalence levels, STH infections affect children's nutrition and play a polarizing role in their immune system.

## 1. Introduction

Soil-transmitted helminths (STH) or geohelminths infect more than 2 billion people worldwide, especially in developing countries located in tropical and subtropical regions. The most prevalent species are* Ascaris lumbricoides* (roundworm),* Trichuris trichiura* (whipworm), and hookworms (*Necator americanus* and* Ancylostoma duodenale*). STH infections account for about 40% of global morbidity due to infectious diseases [1]. Children and women of childbearing age are among the high-risk groups for these parasitic diseases. The morbidity caused by STH infections is highly associated with the size of the worm population residing in the intestines (also known as worm burden). Infections of heavy intensity are more likely to cause impaired physical growth and cognitive development as well as micronutrient deficiencies, including iron-deficiency anaemia [1, 2]. Further, due to their chronic and insidious nature, even light intensity infections may compromise health outcomes. To reduce the morbidity caused by these parasitoses, the World Health Organization (WHO) recommends cyclical (annual or biannual) benzimidazole-based mass drug administration to groups at high-risk of infection, especially pre- and schoolchildren [1]. The basic premise of this strategy is that even though this intervention may not decrease transmission and prevalence, it will in time reduce hosts' worm burden with the ensuing reduction in health impact.

In addition to health impact, STH infections affect the human host in more subtle manner: as extracellular metazoan parasites, helminths trigger type-2 immune response mediated by T-helper type 2 (Th2) cells. This response is typically characterized by expansion of mast cells, eosinophils, basophils, group 2 innate lymphoid cells (ILC-2), and alternatively activated macrophages (AAMs), as well as increased production of Th2 cytokines (e.g., IL-4, IL-5, IL-9, and IL-13) and IgE [3–5].

On the other hand, intracellular pathogens such as viruses, bacteria, protozoa, and fungi elicit a type-1 response, characterized by increased numbers of phagocytic neutrophils and macrophages, as well as cytotoxic CD8^+^ T cells and Th1 cells. Antigen-presenting dendritic cells (DCs) secrete interleukin- (IL-) 12 promoting differentiation of naive CD4^+^ cells, first into null T-helper (Th0) and then into Th1. Via production of interferon-gamma (IFN-*γ*) and IL-2 to a lesser degree, Th1 cells direct, maintain, and enhance the antimicrobial effect of type-1 response [6]. Both subsets of T-helper cells antagonize and negatively regulate each other. Naturally, a healthy resolution of infections relies on a dynamic combination of the two [5, 7].

Chronic helminth infections such as the ones caused by STH exhibit a modified type-2 response, in which a superimposition of regulatory mechanisms exerted over the basic response pattern takes place. This regulation is mainly achieved by the expansion and induction of regulatory T cells (Treg). Increased levels of anti-inflammatory cytokines IL-10 and transforming growth factor beta (TGF-*β*) are hallmarks of this response [4, 8, 9]. It has been suggested that, during chronic helminthiases, this anti-inflammatory network may play a key role in the lower prevalence of allergic disease observed in Th2-skewed populations [7, 10–12]. Conversely, helminth-induced IL-10-mediated immunosuppression has raised concerns for populations in which STH infections coexist with other morbidities such as HIV/AIDS, malaria, and tuberculosis, as clearance for the latter depends on an active and timely type-1 response [8, 13–16].

In this context, successful deworming treatment has been proposed as a practical and inexpensive way to rebalance the immune response [17, 18]. Recent studies have observed that after administration of anthelminthic medication, eosinophil counts [19], IL-10 levels [19–21], and IgE concentrations [17] show a significant decrease from pretreatment values. Moreover, in HIV-1 infected individuals, deworming was shown to promote a significant decrease in plasma HIV RNA levels [18, 22] and CD8^+^ T cells counts [18]. Also, a randomized control trial in Kenya demonstrated a significant increase in CD4^+^ T cell counts after deworming [22]. Other studies, however, found no evidence of this effect [18, 19].

The principal aim of the present study was to characterize the immune profile in schoolchildren infected with geohelminths. Secondary aims involved assessing the health impact and epidemiological determinants of such parasitoses.

## 2. Materials and Methods

### 2.1. Study Population and Ethical Approval

The study was conducted in April 2014 and data were collected from schoolchildren living in Linaca, a rural community of the Municipality of Tatumbla, Department of Francisco Morazán in Honduras. The study was approved by Brock University's Bioscience Research Ethics Board (file number BU 12-262, dated June 27, 2013) as well as by the Research Ethics Board of the Master's Program in Infectious and Zoonotic Diseases, School of Microbiology, National Autonomous University of Honduras, UNAH (file number CEI-MEIZ 01-2013, dated September 23, 2013). Linaca school authorities also approved the study. The school of Linaca participates in the Honduras National Deworming Program, which provides enrolled children with a 400 mg single-dose albendazole annually. At the time of the study, the last deworming round occurred in November 2013. Children who had not received deworming treatment from other sources in the past 3 months were included in the study. Written informed consent was provided by children's parents. Children's oral assents were individually obtained and documented through a child assent form.

### 2.2. Data Collection

Basic demographic and epidemiological data of participant children were obtained using a structured questionnaire during a 5-minute face-to-face interview conducted in Spanish. Body weight and height of participants were measured twice by different researchers and their average was used to calculate the following anthropometric indicators: (a) height-for-age *Z*-score (HAZ), (b) weight-for-age *Z*-score (WAZ), and (c) body-mass-index-for-age *Z*-score (BAZ). These indicators were used to ascertain children's nutritional status as per international parameters: stunted growth (chronic malnutrition), thinness, and underweight, respectively [23].

### 2.3. Stool Collection and Parasite Determination

A same-day single stool sample was provided by each participant the morning of the interview. Samples were kept in portable coolers and examined early afternoon at the laboratory facilities of UNAH's School of Microbiology. The presence of intestinal parasites was assessed using both Kato-Katz and formol-ethyl acetate concentration methods. For the latter, an aliquot was preserved in 10% formalin. Microscopic examination of Kato-Katz smears was done between 30 and 60 min of preparation. Helminth eggs were identified and counted and the number of eggs per gram (epg) was calculated. Infection intensities were classified as light, moderate, or heavy, based on the epg calculations, according to WHO criteria [1]. Diagnostic accuracy was ensured by having 100% of negative and 10% of positive smears read again by a different researcher immediately after the first reading. Formol-ethyl acetate was done one month after sample collection and sediments were observed with dry and immersion oil objective lenses in order to identify helminth and protozoa stages, respectively.

### 2.4. Blood Collection and Hematological Analyses

Blood samples obtained from the cubital vein were collected in 3 mL tubes of each K_2_EDTA and serum separator and clot activator (BD Vacutainer, NJ, USA). The latter were centrifuged within 4 hours and serum was stored at −21°C until immunological analysis. Hematological automated analyses were performed within 4 hours of sample collection and were done with an ABX Pentra 120 (HORIBA-ABX-SAS, Montpellier, France). According to the parameters established in the most recent (2011-2012) Demographic and Health Survey in Honduras (DHS-HN) [24], anaemia was defined as Hb concentration < 12 g/dL. Eosinophilia was defined according to international standards as eosinophil count ≥ 500/*μ*L in peripheral blood. Mild, moderate, or severe eosinophilia were defined if cell counts were 500–1500/*μ*L, >1500–5000/*μ*L, or >5000/*μ*L, respectively [25].

### 2.5. Immunological Biomarkers

Cytokine concentrations in serum samples were measured using a MagPix magnetic beads platform (Luminex xMAP, Austin, TX, USA). Multiplex panels containing magnetic beads covered with fluorescent dyed conjugated to a monoclonal antibody specific for each target molecule were used to quantify Th1/Th2 cytokines (IL-2, IL-6, IL-8, IL-12p70, IFN-*γ*, GM-CSF, TNF-*α*, IL-4, IL-5, and IL-13) and the regulatory cytokine IL-10.

Serum concentrations of total IgE were also quantified using magnetic beads platform but with singleplex kits, Bio-Plex Pro™ Human IgE Isotyping (Bio-Rad laboratories, Inc. Hercules, CA, USA). Hyper-IgE was defined as a serum concentration exceeding 100 IU/mL and was classified as mild (>100–399 IU/mL), moderate (>399–999 IU/mL), or severe (≥1000 IU/mL) [26]. Cytokine and IgE tests were performed according to the manufacturer's instructions (Bio-Rad laboratories, Inc. Hercules, CA, USA).

### 2.6. Statistical Analyses

Descriptive statistics were used to characterize the study population. Point prevalence with 95% confidence intervals (95% CI) was calculated for overall STH infections and for each parasite species, as well as for mixed STH infections (i.e., infected with two or more species). Anthropometric indicators were calculated using the WHO AnthroPlus software ver. 1.04 (WHO). Associations between nutritional indicators and parasite infections were explored using both univariate and multivariable logistic regression models. Unadjusted and adjusted odds ratio (OR and adj. OR) with 95% CI were determined. Due to the non-Gaussian distribution of the immunological markers, nonparametric methods were used as follows. Geometric means and 95% CI were calculated for each marker. Relationships were evaluated through Spearman's rank correlation coefficient. Kruskal-Wallis test was used to assess differences among groups, followed by Dunn's test to investigate individual group differences. All statistical analyses were conducted using Stata 13 (StataCorp LP, TX, USA) and the level of significance was defined as *p* < 0.05.

## 3. Results

### 3.1. Characteristics of the Study Population

Table 1 summarizes the characteristics and parasitological findings in the study population. A total of 225 schoolchildren attending grades 1 to 6 (age 6–13) participated in the study. All provided stool samples and all but one child agreed to provide blood samples. Most of the children lived in households with piped water (88%) and flushing toilet and/or latrines (98%) but 40% lived in households with earthen floor.

### 3.2. Parasitological Findings

Qualitative Kato-Katz results were 100% correlated with those of formol-ethyl acetate. No cases of* S. stercoralis* or hookworm infections were found with either technique but one case of* Taenia solium* was identified by the presence of gravid proglottids in the stool sample. Among pathogenic protozoa,* Giardia intestinalis* and* Entamoeba histolytica/dispar* were present in 4% and 9% of the samples, respectively. Additionally, one case of* Cyclospora cayetanensis* was identified.

The overall STH prevalence was 30% (95% CI = 24.1–36.1). Prevalence for* T. trichiura* and* A. lumbricoides* was 22.2% (95% CI = 17.2–28.2) and 20.4% (95% CI = 15.6–26.2), respectively. Mixed STH infections represented 43% of all infections observed. While the majority (84%) of trichuriasis cases were light intensity, about one-third (32.6%) of ascariasis cases were moderate-to-heavy intensity (Table 1). Only 20% of moderate-to-heavy infections with both* A. lumbricoides *and* T. trichiura* occurred as single infections. In the multivariable analysis, age was not associated with parasitism. Ascariasis was associated with sex and some household conditions of participating children. Almost three-quarters (71.7%) of ascariasis cases occurred in boys and they had almost three times the odds of having* A. lumbricoides* infections compared to girls (adj. OR = 2.82, 95% CI = 1.34–5.94, and *p* = 0.006). Children living in households with earthen floor had twice the odds of helminthic infections (adj. OR = 2.29, 95% CI = 1.15–4.55, and *p* = 0.018). Conversely, children with access to piped water in their households showed 64% reduced odds of ascariasis (adj. OR = 0.36, 95% CI = 0.14–0.93, and *p* = 0.035).* T. trichiura* infections were not significantly associated with any of the abovementioned risk factors.

### 3.3. Nutritional Status

About 12% of the children were overweight, and this condition was significantly higher in boys than girls (73% versus 27%, *p* = 0.036); only one of these children was stunted. Stunted growth was observed in 9.8% of the studied children. Multivariable logistic models controlling for age and sex were constructed, and adjusted OR were calculated. In this population, stunting was significantly associated with the age of participants as well as with trichuriasis. Per every year of age, the odds of stunting increased in 34% (adj. OR = 1.34, 95% CI = 1.03–1.73, and *p* = 0.025). Similarly, children harbouring* T. trichiura* infections had almost four times the odds of being stunted (adj. OR = 3.93, 95% CI = 1.03–14.93, and *p* = 0.045) when compared with children without STH infection. Further, children harbouring moderate-to-heavy trichuriasis had an additional 70% increased odds of being stunted (adj. OR = 6.64, 95% CI = 1.19–37.09, and *p* = 0.031).

### 3.4. Eosinophilia and IgE Levels

**Figure 1:**
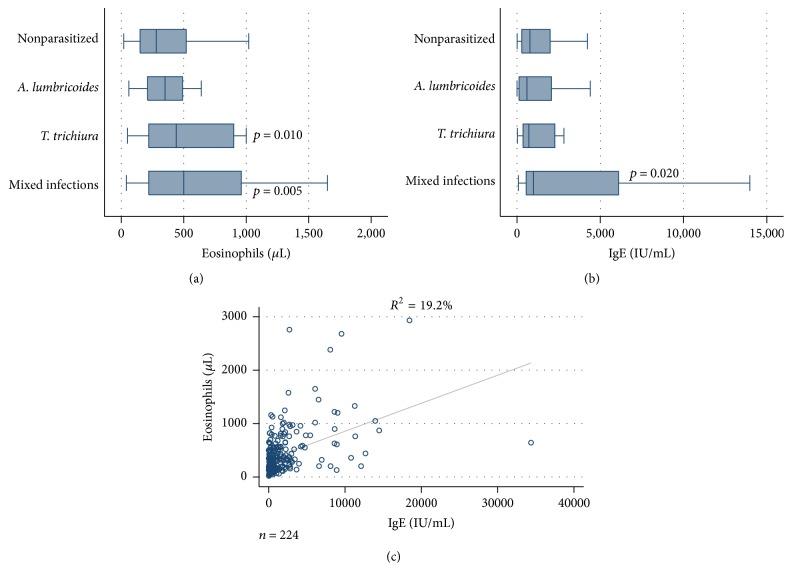
Association between STH infections and eosinophils count and serum levels of IgE in studied children. Higher counts in eosinophils were significantly associated with trichuriasis and mixed infections (a). Higher total IgE levels were associated with mixed infections (b). Moderate positive correlation of eosinophils count and total IgE levels (c).

**Figure 2:**
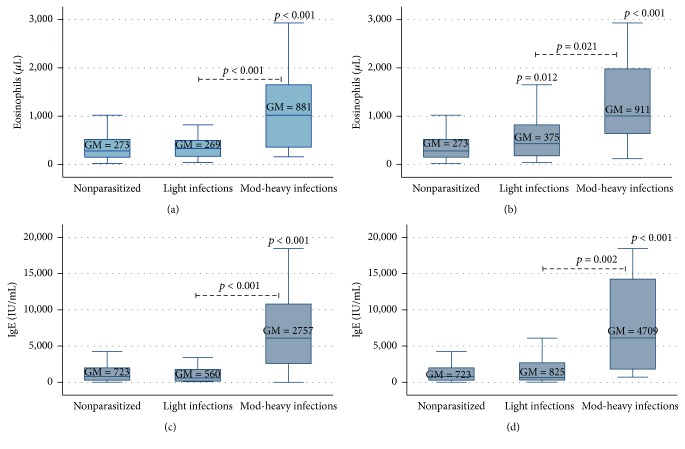
Association between STH infection intensity and eosinophils count and serum levels of IgE in studied children. Moderate-to-heavy infections of* A. lumbricoides* (a, c) and* T. trichiura* (b, d) were associated with higher eosinophils count and total IgE levels. Geometric means (GM) are depicted in their respective boxes.

Figures 1 and 2 depict the variation observed in eosinophils count (cells/*μ*L) and serum levels of IgE by STH infection and infection intensity. The geometric means and 95% CI of these parameters by STH infection are shown in Table 2. The overall prevalence of eosinophilia was 31.7% (95% CI = 25.9–38.1), and mild eosinophilia accounted for the vast majority (91.5%) of cases. No severe cases of eosinophilia were identified.

Multivariable logistic models found no association between eosinophilia and sex or age of the studied children. Significantly higher mean counts of eosinophils were found in children infected with* T. trichiura* alone or in those harbouring mixed infections compared to nonparasitized children. A significant association was found between eosinophilia and STH infection and infection intensity. Children harbouring mixed infections had 2.5-fold increased odds of having eosinophilia when compared to nonparasitized children (adj. OR = 2.59, 95% CI = 1.13–5.90, and *p* = 0.023). Similar increased odds were observed in children with single infection by* T. trichiura*, although this effect was only marginally significant (adj. OR = 2.48, 95% CI = 0.98–6.26, *p* = 0.054). Eosinophilia was positively correlated with intensity of infection by both* A. lumbricoides* and* T. trichiura* (*r*_*s*_ = 0.26, *p* < 0.001 and *r*_*s*_ = 0.33, *p* < 0.001, resp.). Children with moderate-to-heavy ascariasis had five times the odds of presenting eosinophilia (adj. OR = 5.23, 95% CI = 1.69–16.12, and *p* = 0.004) compared to their nonparasitized counterparts. Likewise, moderate-to-heavy trichuriasis was associated with a 7-fold increased odds of eosinophilia (adj. OR = 7.19, 95% CI = 1.41–36.70, and *p* = 0.018).

The overall prevalence of hyper-IgE was 90.2% (95% CI = 85.5–93.5). More than half of these cases (51.0%) were severe, whereas mild and moderate hyper-IgE accounted for 24.3% and 24.7% of the cases, respectively. No significant correlation was found between IgE levels and sex or age of participant children. Compared to those without STH infections, children with mixed STH infections had significantly higher mean levels of IgE (Table 2). There was a moderate positive correlation between IgE serum levels and eosinophils count (*r*_*s*_ = 0.43, *p* < 0.001). Similarly, IgE levels were positively correlated with intensity of infection of both* A. lumbricoides* and* T. trichiura*. Children with moderate-to-heavy ascariasis or trichuriasis had significantly higher mean values of IgE compared to those uninfected or with only light infections (*p* < 0.001) (Figure 2).

### 3.5. Serum Cytokines Levels

**Figure 3:**
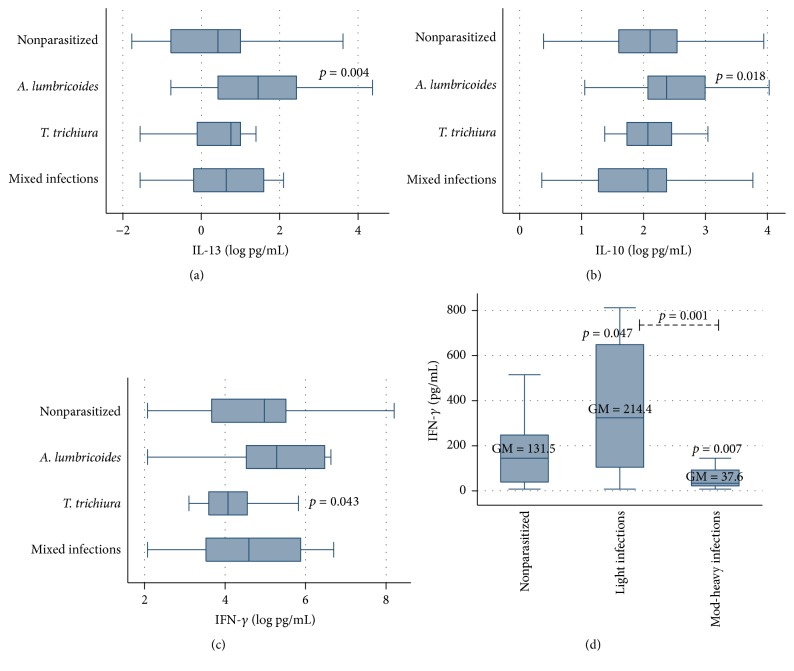
Association between STH infection and cytokines in studied children.* A. lumbricoides* infections were associated with higher levels of IL-13 (a) and IL-10 (b), whereas lower levels of IFN-*γ* were associated with* T. trichiura *infections (c) and moderate-to-heavy ascariasis (d). Geometric means (GM) are depicted in their respective boxes.

Th1 and Th2 representative cytokines were determined in serum samples. Table 2 summarizes the geometric mean values and 95% CI of these cytokines by STH infection. In general, cytokine mean concentrations did not differ significantly when comparing the parasitized with the nonparasitized group. However, children with single infections by* A. lumbricoides* had significantly higher levels of IL-10 and IL-13 compared to children without infection, with single* T. trichiura* infections or with mixed infections (*p* = 0.018 and *p* = 0.004, resp.). Spearman's rank correlation coefficients showed a significant, although weak, negative correlation between IL-10 levels and age (*r*_*s*_ = −0.23, *p* < 0.001). IL-10 levels were not significantly correlated with infection intensity, whereas for IL-13 a weak but significant positive correlation with* A. lumbricoides* infection intensity was found (*r*_*s*_ = 0.22, *p* = 0.012). Significantly lower values of IFN-*γ* were found in children with single infections by* T. trichiura* (*p* = 0.043) and these values showed a weak, negative correlation with* T. trichiura* infection intensity, although this correlation was not statistically significant (*r*_*s*_ = −0.17, *p* = 0.069) (Figure 3).

**Figure 4:**
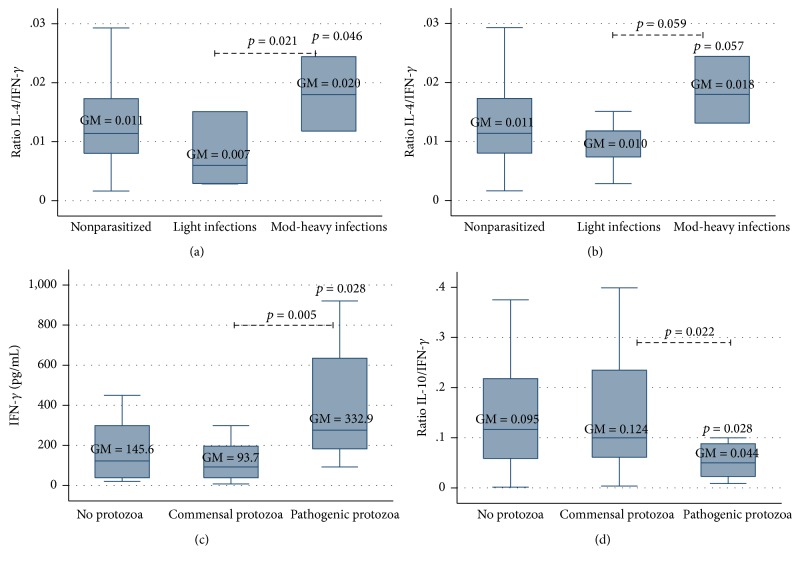
Association between STH infection intensity and Th2/Th1 cytokines ratio in studied children. Higher IL-4/IFN-*γ* ratios were associated with moderate-to-heavy ascariasis (a) and trichuriasis (b), although marginally significant in the latter. In children without STH infections, pathogenic protozoa were associated with higher levels of IFN-*γ* (c) and lower IL-10/IFN-*γ* (d). Geometric means (GM) are depicted in their respective boxes.

On the other hand, IFN-*γ* values were significantly negatively associated with* A. lumbricoides* infection intensity (*p* = 0.007). Children with moderate-to-heavy ascariasis had lower IFN-*γ* mean values (37.6 [15.7–90.0] pg/mL) compared with those having no or light infections (131.5 [98.4–175.8] pg/mL and 214.4 [99.0–464.4] pg/mL, resp.) (Figure 3). Th2/Th1 ratios, as well as IL-4/IFN-*γ* and IL-10/IFN-*γ*, were also calculated; significantly higher IL-4/IFN-*γ* ratios were obtained in children with moderate-to-heavy infections by* A. lumbricoides* (0.020 [0.010–0.042]), compared to those without (0.011 [0.08–0.014]) or light infections (0.007 [0.002–0.022]) (*p* = 0.046 and *p* = 0.021, resp.) (Figure 4). A very similar pattern in IL-4/IFN-*γ* ratio was found for* T. trichiura* infection intensity, although differences were only marginally significant (*p* = 0.057 and *p* = 0.059, resp.). Conversely, no significant differences in IL-10/IFN-*γ* ratios were observed.

Finally, the potential effect of pathogenic protozoa in children's immune response was assessed but this assessment was done only in children without STH infections. It was found that IFN-*γ* mean values were significantly higher in children harbouring pathogenic protozoa (i.e.,* G. intestinalis* and/or* E. histolytica/dispar*) compared to those infected with commensals only or to those with no protozoa at all (332.9 [146.7–755.8] pg/mL, 93.7 [60.9–144.2] pg/mL, and 145.6 [93.8–225] pg/mL, resp.). IL-10/IFN-*γ* ratios were significantly lower in children infected with pathogenic protozoa when compared to those uninfected or infected with commensals only (0.044 [0.019–0.105], 0.095 [0.062–0.144], and 0.124 [0.072–0.214], resp.) (Figure 4). No statistically significant associations could be established between protozoa infection and eosinophil counts, IgE levels, or the remaining cytokines studied.

## 4. Discussion

The parasitological findings of this study demonstrate a moderately high (30%) overall STH prevalence and specific prevalence for* T. trichiura* and* A. lumbricoides* of about 20% among schoolchildren living in Linaca. School teachers reported that the school is reached by the national deworming program and thus receives a single-dose 400 mg albendazole tablet per child on a yearly basis. No records are kept at the school in terms of deworming tablets intake and no parasitological baseline data have been obtained during governmental surveys (data reviewed in [27]). To our knowledge, prior to the present study, two parasitological surveys had been undertaken in the study community. In 1985, a study investigating causes of diarrhea in Honduras included a small number of preschoolchildren and found prevalence of 48% and 30% for* A. lumbricoides* and* T. trichiura*, respectively [28, 29]. More recently, a study involving schoolchildren determined prevalence of 64% for* A. lumbricoides* and 46% for* T. trichiura* (Canales et al. 2008, unpublished). Compared to the STH prevalence found in the present study, it appears that there has been an important decrease in STH prevalence among Linaca's children. Due to insufficient data on deworming, we did not attempt to identify if this strategy has served an important function in decreasing STH prevalence or at least worm burden. Thus, whether or not deworming has contributed to STH prevalence reduction remains an open question. Our findings, however, show that improved household conditions were strong protective factors for ascariasis. In this case, there might be a synergy between the built environment and anthelminthic medication, which may not be sufficient to protect against* T. trichiura* infection as one dose of ALB treatment is largely ineffective to clear this parasite.

Even though a similar proportion of children were found with either excess weight (12%) or chronic malnutrition (stunted growth, ~10%), the latter was strongly associated with parasitism. Similarly, despite the fact that trichuriasis prevalence was at a moderate level and that the majority of infections were light, a significant association was documented between stunting and both* T. trichiura* infection and infection intensity. This finding aligns with observations from other studies conducted in Honduras [30], Ethiopia [31], Mexico [32], and Brazil [33, 34].

The concerning association between trichuriasis and chronic malnutrition, along with the fact that* T. trichiura* has been found consistently as the most prevalent geohelminth in Honduras [27, 35, 36], suggests that (i) a reexamination of the current deworming guidelines are necessary; (ii) improvements in sanitary conditions and health education are indispensable components of STH control initiatives; and (iii) it is important to monitor for potential benzimidazole resistance in this parasite [37].

The prevalence of eosinophilia was high among the studied children. It has been shown that, in developing countries, eosinophilia is most commonly induced by tissue-invasive parasites, particularly helminths [25, 38]. STH infections have been largely associated with eosinophilia, especially in early stages of infection, when larval migration occurs. The association between eosinophilia and STH in the present study is consistent with results from previous work conducted in Honduras [39, 40] and other countries such as Brazil [41, 42], Philippines [43], Indonesia [44], and Spain [26].

Whereas other causes of eosinophilia cannot be ruled out, it is likely that, in this particular group of children, such eosinophilia was reactive (i.e., secondary to an external stimulus) and most likely associated to STH infection. This inference is supported by data contained in Linaca's Health Centre morbidity report for 2014, which shows that <2% of children's visits were due to asthma or allergic dermatitis (Linaca Health Care Centre 2014 report, unpublished). Moreover, a comprehensive socioeconomic and life conditions study conducted in the municipality of Tatumbla, where Linaca is located, contained no reference to allergic conditions being a health issue among inhabitants. Rather, it revealed that childhood acute respiratory infections accounted for >50% of visits to the health centre [45].

The use of eosinophilia as a biomarker for helminthiases remains without consensus. As a predictor of current helminthic infections, some authors consider eosinophilia's predictive value very limited [38], while others regard it as a suitable indicator of helminthiases in people from tropical and subtropical areas [26, 42, 44, 46]. Our data support the latter since eosinophilia correlated with both STH infection and intensity. Future studies should investigate eosinophilia at the individual level in order to better understand this dynamic relationship.

Our study revealed strikingly high prevalence of hyper-IgE among the studied children; it also highlighted a significant association between STH infections and increased total IgE levels. Such findings are consistent with observations in studies conducted in Nigeria [47, 48], Ecuador [49], Venezuela [50], Spain [26], and Brazil [41, 51, 52]. Helminth-induced IgE is mostly characterized by a nonspecific polyclonal stimulation with only a small fraction of parasite-specific IgE. However, it is the parasite-specific IgE which plays an important role in helminth clearance as well as in preventing reinfection [48, 50, 53–55]. In a study of Venezuelan children, Hagel and collaborators demonstrated that total IgE levels were inversely correlated with parasite-specific IgE levels. They also showed that reinfection with* A. lumbricoides* was significantly associated with high pretreatment total IgE but low parasite-specific IgE levels [50].

Another interesting finding in the present investigation was the significant association observed between high total IgE levels and intensity of infection (i.e., worm burden). Other researchers have made the same observation [49, 51, 56]. Some studies have shown that specific IgE levels reflect the parasite infection intensity; therefore, higher specific antibody levels would be expected in lightly infected individuals when compared with those heavily infected [57]. Total IgE levels in the studied children were significantly positively correlated with eosinophils counts; this finding is not surprising due to the ability of IgE to induce a cytotoxic response against helminthic parasites mediated by mast cells and eosinophils [55].

It has been demonstrated that helminth infections strongly induce an immune response involving elevated Th2 cytokines (i.e., IL-4, IL-5, IL-9, and IL-13), IgE, IgA, eosinophilia, and mucus secretion [58–60]. Helminth-caused tissue damage triggers the response by releasing danger-associated molecular patterns (DAMPs) and cytokine alarmins, particularly IL-25 and IL-33. These alarmins promote the activation of basophils and innate lymphoid cells (ILC-2) to support a type-2 innate immune response needed to signal the type-2 adaptive immunity. In this context, large quantities of IL-5 and IL-13 are primarily produced by ILC-2 cells. IL-5 promotes the eosinophils differentiation and activation which promote IL-4 production, the main primer of CD4^+^ Th2 cells activation and expansion, with further release of IL-4, IL-5, and IL-13 by this type of cells [3, 60–62]. In this environment, IL-4 and IL-13 induce AAMs, which mainly promote tissue repair and fibrosis by expression of different markers such as arginase-1, Ym1, Ym2, and RELM-*α*. These AAMs also express regulatory IL-10 and TGF-*β* able to downregulate inflammatory response [3–5, 9, 61–63].

Our study could not find a significant correlation between IL-5 and eosinophilia. Instead, the majority of children showed either low or nondetectable values of this cytokine. This was an unexpected finding since IL-5 is essential for eosinophilic differentiation, proliferation, and activation. There are a few potential explanations for this finding. Since the participant children have probably experienced repeated STH infections since they were much younger, their immune response may have already changed to a modified Type 2 response, where inflammatory Th2 shifted to an attenuated phenotype characterized by the shutdown of effector cytokines such as IL-5, retention of IL-4, and reinforcement of anti-inflammatory cytokines such as IL-10 and/or TGF-*β* [4, 9]. Instead, as recently determined by Fulkerson and colleagues, an alternative IL-5-independent pathway for promoting eosinophilia might be in place. This pathway would contribute to persistent eosinophil differentiation and survival even after a complete IL-5 withdrawal [64]. Finally, periodic deworming followed by reinfection along several years is likely disturbing the antiparasitic immune responses as described in the literature.

We found that IL-13 levels were significantly increased in children infected with* A. lumbricoides* and that these levels were positively correlated with worm burden. IL-13 is a Th2 pleiotropic cytokine with an important function in the intestinal epithelia, controlling the rate of transit of epithelial cells toward the outmost layer and promoting a harsh environment that might help in gut parasite expulsion (i.e., increased luminal fluids, mucus, from goblet cells, increased edema, and muscle contractility) [7, 60, 61]. Gallo and colleagues demonstrated that IL-13 may also be produced by Th1 and Th17 cells, suggesting that this cytokine can have either a pro- or anti-inflammatory effect, depending on the environment [65]. Nevertheless, demonstrating an association between STH and IL-13 overproduction has not been achieved by other research groups in India [66] and Brazil [67]. Some studies in Cameroon [68, 69] found high levels of IL-13 associated with lower intensity of infection, but the present study did not establish such association.

IL-10 is the anti-inflammatory cytokine by excellence. It can be produced by diverse type of cells such as macrophages, dendritic cells (DC), B cells, and various subsets of CD4^+^ and CD8^+^ T cells. Regardless of the type of infection, IL-10 limits Th1 and Th2 effector responses by suppressing function of macrophages and DCs. The timing, site of its production, and strength may favor (a) simultaneous pathogen clearance and suppression of downstream pathologies; (b) potential benefit to both the host and the pathogen (limiting pathology and allowing persistent infection); (c) severe tissue damage; or (d) overwhelming infection [70, 71]. In our study, statistically significant higher IL-10 values were found in children with ascariasis, although they did not correlate with infection intensity of this parasite. Similar findings have been frequently reported by other researchers [66, 69, 72–74], with some exceptions [47]. We also found a negative correlation between children's age and IL-10 levels. This is congruent with previous studies conducted in a different community in Honduras [75] as well as with other studies in Nigeria [73] and Cameroon [68, 76]. This might be due to altered immune modulation provoked by deworming treatment. Although its efficacy is species-dependent, albendazole generally reduces the duration and/or intensity of helminth infections [75].

In our study, IFN-*γ* levels were significantly inversely associated with intensity of infection by* A. lumbricoides.* It is well known that IFN-*γ* is downregulated by type-2 cytokines making our finding consistent with the literature. IFN-*γ* is the most important type-1 cytokine and is produced by a diverse type of cells from both innate and adaptive immune arms such as natural killer (NK), CD4^+^ Th1, CD8^+^ Tc, and eosinophils. It interacts with macrophages to activate direct antimicrobial and antitumor mechanisms as well as upregulating antigen processing and presentation pathways [77].

The overall interpretation of the different cytokines, their levels, and relationships with helminthic infections is an interesting challenge. In moderate-to-heavy ascariasis, we observed a distinctive type-2 immune response. This response was characterized by upregulation of IL-13 and downregulation of IFN-*γ*, as well as by significantly higher IL-4/IFN-*γ* ratio and IgE levels. Results from* T. trichiura* infections were not as well-defined as those from ascariasis. Nonetheless and similarly to* A. lumbricoides*, infections of high intensity were associated with lower values of IFN-*γ* and higher IL-4/IFN-*γ* ratios and IgE levels. These findings confirm that a type-2 response against this parasite was also displayed in our study population.

As mentioned earlier, a type-1 immune response is needed for the control and clearance of intestinal protozoa such as* Entamoeba histolytica*,* Cryptosporidium* spp., and* Giardia intestinalis*. Although, particularly in the case of* G. intestinalis*, mixed Th1-Th2 responses have been reported, human and animal studies have shown that increased levels of IFN-*γ* play a crucial role in orchestrating the immune response and appear to be one of the main signatures in individuals with these parasitoses [78–81]. This description is in agreement with our findings of significantly higher (up to 3-fold) levels of IFN-*γ* found in children harbouring* G. intestinalis* and/or* E. histolytica/dispar* without the immune influence of STH infections (i.e., negative for helminthic infections). Moreover, the significantly lower IL10/IFN-*γ* ratios observed in this group of children indicate a Th1-biased response. Additionally, in a 6-year longitudinal study in Bolivia, Blackwell and colleagues described an antagonist relationship between* G. intestinalis* and geohelminths where* giardiasis* was less likely to be present in helminth-infected individuals. Likewise, they also found that infection with helminths was less likely for individuals with giardiasis [82]. Our data, though cross-sectional, support these observations: none of the children infected with* Giardia intestinalis* had ascariasis and only 10% of them had concomitant trichuriasis. Since clearance and protective immunity against* G. intestinalis* require also Th2 cytokines and antibodies, IgA, IgG, and IgE [78, 79, 81], a plausible scenario has been suggested: helminth-induced Th2 response might provide cross-immunity against this protozoan. We believe that the STH/*G. intestinalis* interaction is worth investigating.

A proper interpretation of our findings must be done considering the limitations of the present study. Firstly, its cross-sectional nature only allows establishing associations but no causality and, therefore, strong inferences cannot be made. Secondly, we did not obtain a complete medical history from participating children or an accurate deworming history. This is important as a variety of unmeasured confounding variables (e.g., bacterial and viral infections, autoimmune diseases, asthma, and other allergic diseases, as well as environmental and genetic factors) may have contributed to skew the Th1/Th2 balance among research participants [6]. Thirdly, we measured circulating levels of cytokines, which may not be an accurate representation of local immune responses in the gut milieu. Notwithstanding, the study has several strengths: an adequate sample size, determination of both helminthic and protozoal infections, and the use of highly sensitive and accurate methodology for biomarkers analysis.

## 5. Conclusion

This is the first study providing a comprehensive immune profile in Honduran children infected with geohelminths or pathogenic protozoa. Our study shows that higher intensities of STH infections are associated with a polarized Th2 response, characterized by reduced proinflammatory and increased regulatory cytokines, eosinophilia, and hyper-IgE. It also demonstrates that an enhanced Th1 response is elicited by pathogenic protozoa. The interpretation of the host's immune response against helminth represents challenging undertaking due to the high complexity of multiple host-parasite interactions and potential unmeasured confounders. Pivotal to characterizing and understanding the immune response is obtaining a meticulous clinical history from study participants, their deworming history, and very importantly the existence of multiple infections including pathogenic protozoa. Further, the role of the intestinal microbiota and its interplay with the host and its parasites can no longer be ignored. Longitudinal studies are required to better characterize such immune responses.

## Figures and Tables

**Table 1:** Characteristics and parasitological findings of the study population (*n* = 225).

Characteristics	*n* (%)
Age—mean (SD)	8.96 (1.8)
Girls	104 (46.2)
*Household conditions*	
Earthen floor (complete or partial)	91 (40.4)
Type of sanitary facility available	
None	5 (2.2)
Latrine	115 (51.1)
Toilet	105 (46.7)
Access to piped water	198 (88.0)
*Nutritional indicators*	
Height-for-age *Z*-score (HAZ)—mean (SD)	−0.76 (1.03)
Weight-for-age *Z*-score (WAZ)—mean (SD) (*n* = 155)^a^	−0.19 (1.05)
Body mass index-for-age *Z*-score (BAZ)—mean (SD)	0.22 (0.93)
Stunting (<−2 *SD* HAZ)	22 (9.8)
Underweight (<−2 *SD* WAZ)^a^	3 (1.9)
Thinness (<−2 *SD* BAZ)	1 (0.4)
Overweight (>1 *SD* & ≤2 *SD* BAZ)	26 (11.6)
Obesity (>2 *SD* BAZ)	11 (4.9)
Hemoglobin (g/dL)—mean (SD) (*n* = 224)^b^	13.7 (0.8)
Presence of anemia^b^	8 (3.6)
*Parasitic profile*	
Overall prevalence of STH infections	67 (29.8)
Overall prevalence of *Ascaris lumbricoides*	46 (20.4)
Overall prevalence of *Trichuris trichiura*	50 (22.2)
Overall prevalence of Hookworms	0 (0.0)
Single *Ascaris lumbricoides* infections (*n* = 67)	17 (25.4)
Single *Trichuris trichiura* infections (*n* = 67)	21 (31.3)
Mixed infections (*n* = 67)	29 (43.3)
Moderate-to-heavy infections by *Ascaris lumbricoides *(*n* = 46)	15 (32.6)
Moderate-to-heavy infections by *Trichuris trichiura *(*n* = 50)	8 (16.0)

STH: soil-transmitted helminth.

^a^Not calculated in children older than 10 years of age.

^b^One child did not agree on providing blood sample.

**Table 2:** Geometric means (95%CI) of immunological markers determined in schoolchildren by STH infection status (*n* = 224)^a^.

Parameter	Nonparasitized	Ascariasis	Trichuriasis	Mix infections
	*n* = 178	*n* = 17	*n* = 21	*n* = 29
Eosinophils (cells/*µ*L)	273.5 (240.7–310.8)	320.8 (229.7–448.0)	410.1 (277.1–606.9)^*∗*^	448.9 (292.2–689.5)^*∗∗*^
IgE (IU/mL)	722.9 (573.0–912.1)	443.9 (159.1–1238.2)	726.1 (352.4–1495.9)	1463.7 (805.9–2658.4)^*∗*^
IL-2 (pg/mL)	26.1 (21.0–32.4)	34.7 (19.7–61.2)	24.1 (16.0–36.3)	21.3 (14.4–31.6)
IL-4 (pg/mL)	1.2 (0.8–1.7)	0.7 (0.1–5.4)	0.9 (0.1–5.0)	0.8 (0.2–2.4)
IL-5 (pg/mL)	10.9 (4.6–25.8)	17.1 (4.4–66.6)	11.8 (9.4–14.9)	5.1 (1.2–21.7)
IL-6 (pg/mL)	13.7 (11.2–16.8)	16.1 (7.9–32.8)	10.8 (5.9–19.7)	9.8 (6.4–14.8)
IL-8 (pg/mL)	107.2 (91.8–125.2)	105.8 (65.5–170.9)	91.8 (61.1–138.1)	99.3 (71.1–138.7)
IL-10 (pg/mL)	8.5 (7.1–10.1)	15.3 (6.8–34.6)^*∗*^	7.1 (4.8–10.5)	6.6 (4.9–8.8)
IL-12p70 (pg/mL)	9.5 (7.5–12.1)	15.3 (5.7–41.1)	7.4 (5.2–10.5)	6.3 (4.1–9.8)
IL-13 (pg/mL)	1.2 (0.9–1.5)	4.7 (1.5–15.0)^*∗∗*^	1.3 (0.6–2.7)	1.9 (0.9–3.9)
IFN-*γ* (pg/mL)	131.5 (98.4–175.8)	162.9 (49.7–533.2)	70.1 (35.6–138.0)^*∗*^	95.5 (38.7–235.7)
GM-CSF (pg/mL)	55.1 (43.9–69.0)	72.8 (38.8–136.5)	45.5 (22.1–93.4)	45.0 (23.0–87.9)
TNF-*α* (pg/mL)	8.2 (6.7–10.2)	10.4 (3.7–28.9)	9.1 (5.1–16.0)	6.4 (3.8–10.7)

STH: soil-transmitted helminth.

^a^One child did not agree on providing blood sample.

^*∗*^ *p* < 0.05.

^*∗∗*^ *p* < 0.01.
